# Assessing the role of toll-like receptor in isolated, standard and enriched housing conditions

**DOI:** 10.1371/journal.pone.0222818

**Published:** 2019-10-24

**Authors:** Tahani K. Alshammari, Hajar Alghamdi, Thomas A. Green, Abdurahman Niazy, Lama Alkahdar, Nouf Alrasheed, Khalid Alhosaini, Mohammed Alswayyed, Ramesh Elango, Fernanda Laezza, Musaad A. Alshammari, Hazar Yacoub

**Affiliations:** 1 Department of Pharmacology and Toxicology, Pharmacy College, King Saud University, Riyadh, Saudi Arabia; 2 Pharmacology & Toxicology Graduate Program, Pharmacy College, King Saud University, Riyadh, Saudi Arabia; 3 Department of Pharmacology and Toxicology, University of Texas Medical Branch, Galveston, TX, United States of America; 4 Prince Naïf Bin Abdul-Aziz Health Research Center, King Saud University, Riyadh, Saudi Arabia; 5 Department of Pathology and Laboratory Medicine, College of Medicine, King Saud University Medical City, King Saud University, Riyadh, Saudi Arabia; 6 Stem Cell Unit, Department of Anatomy, College of Medicine, King Saud University, Riyadh, Saudi Arabia; Radboud University Medical Centre, NETHERLANDS

## Abstract

Depression is a common psychiatric disorder that has been poorly understood. Consequently, current antidepressant agents have clinical limitations. Until today, most have exhibited the slow onset of therapeutic action and, more importantly, their effect on remission has been minimal. Thus, the need to find new forms of therapeutic intervention is urgent. The inflammation hypothesis of depression is widely acknowledged and is one that theories the relationship between the function of the immune system and its contribution to the neurobiology of depression. In this research, we utilized an environmental isolation (EI) approach as a valid animal model of depression, employing biochemical, molecular, and behavioral studies. The aim was to investigate the anti-inflammatory effect of etanercept, a tumor necrosis factor-α inhibitor on a toll-like receptor 7 (TLR 7) signaling pathway in a depressive rat model, and compare these actions to fluoxetine, a standard antidepressant agent. The behavioral analysis indicates that depression-related symptoms are reduced after acute administration of fluoxetine and, to a lesser extent, etanercept, and are prevented by enriched environment (EE) housing conditions. Experimental studies were conducted by evaluating immobility time in the force swim test and pleasant feeling in the sucrose preference test. The mRNA expression of the TLR 7 pathway in the hippocampus showed that TLR 7, MYD88, and TRAF6 were elevated in isolated rats compared to the standard group, and that acute treatment with an antidepressant and anti-inflammatory drugs reversed these effects. This research indicates that stressful events have an impact on behavioral well-being, TLR7 gene expression, and the TLR7 pathway. We also found that peripheral administration of etanercept reduces depressive-like behaviour in isolated rats: this could be due to the indirect modulation of the TLR7 pathway and other TLRs in the brain. Furthermore, fluoxetine treatment reversed depressive-like behaviour and molecularly modulated the expression of TLR7, suggesting that fluoxetine exerts antidepressant effects partially by modulating the TLR7 signaling pathway.

## 1. Introduction

Major depression is psychotic mood disorder represented by different symptoms such as mood disturbance, sleep dysregulation, and decreased appetite [1, 2]. According to the world health organization, more than 300 million people globally of all ages suffer from depression. Also, it is the leading cause of disability which accounts for 7.4% of total disability-adjusted life year worldwide and is a significant contributor to the overall global burden of disease. In severe cases, depression can lead to suicide [3]. Today, the existing first-line pharmacological treatments (SSRIs and SNRIs) are inefficient. Studies show that one out of 7 patients gains a positive outcome. Evidence shows that exposure to specific psychological experiences, including stress-induced diseases, is associated with variation in immune parameters. A recent study indicated that innate immune responses are highly engaged after stressful events and during the depressive episode. Furthermore, a depressed patient shows increased circulating peripheral cytokines [4].

The inflammation hypothesis of depression is well developed. This theory aims to understand the relationship between the function of the immune system and its contribution to the neurobiology of depression. More recently, an abundance of experimental evidence suggests that activation of innate immune mechanisms, especially tumor necrosis factor alpha, proinflammatory cytokines, and C-reactive protein, may contribute to psychiatric disease pathology such as depression [5, 6]. Additionally, increased expression of a variety of innate immune genes and proteins, including IL-1β, IL-6, TNF, Toll-like receptor 3 (TLR3) and TLR4, has been found in post-mortem brain samples from individuals with depression that died by suicide [7]. Moreover, mounting evidence indicates that inflammatory cytokines are associated with resistance to monoaminergic treatment[8, 9]. Further evidence also shows that inflammatory cytokines can cause behavioral alterations. 20% to 50% of patients receiving chronic IFN-alpha therapy for the treatment of infectious diseases or cancer develop clinically significant depression [10, 11].

The toll-like receptor (TLR) family was discovered in 1997 by Dr. Charles Janeway as a Toll homolog in human monocytes, namely TLR4. Members of TLR family are expressed in a variety of cell types including immune cells, muscle cells, heart, and intrinsic central nervous systems (CNS) cell types such as neurons, astrocytes, and microglia[12–14]. Several studies have identified a relationship between depression and upregulation of TLRs in depressed brain. Both TLR3 and TLR4, have been found in post-mortem brain samples from individuals with depression that died by suicide that suffered from depression [7, 10]. Increasingly, TLRs are gaining interest in the field of neuroscience, including their potential roles in the neurobiology of brain disorders [4, 15]. For instance, the possible role of TLR-4 in the regulation of stress-induced neuroinflammatory signals were analyzed. Although a study has shown that Toll-like receptor 7 (TLR7) transcript level is elevated in a genetically modified depressive mouse model [3], the exact role of TLR7 and its pathway components have not yet been investigated.

We hypothesized that TLR signaling is altered in hippocampus and nucleus accumbens (NAc) in an animal model of depression (environmental isolation vs. enrichment), that TLR signaling is involved in the pathology of depression and could be altered after chronic administration of Fluoxetine in rats. Moreover, we used etanercept treatment as a control for anti-inflammatory effects.

## 2. Material and methods

### Animals

Adult male Wistar rats (150–175 g) were obtained from the Animal Care Centre at the College of Pharmacy, King Saud University, Riyadh, Saudi Arabia. Rats were housed in 12-hour regular light/dark cycle) and temperature (25±1°C) with free access to food and water. Rats were allowed to adapt to the laboratory environment for one week before the start of the experiments. All behavioral tests were conducted between 08:00 a.m. and 12:00 p.m. The Experimental Animals Ethics Committee Acts of King Saud University approved this study (Ref. NO.: KSU-SE-18-20).

**Fig 1:**
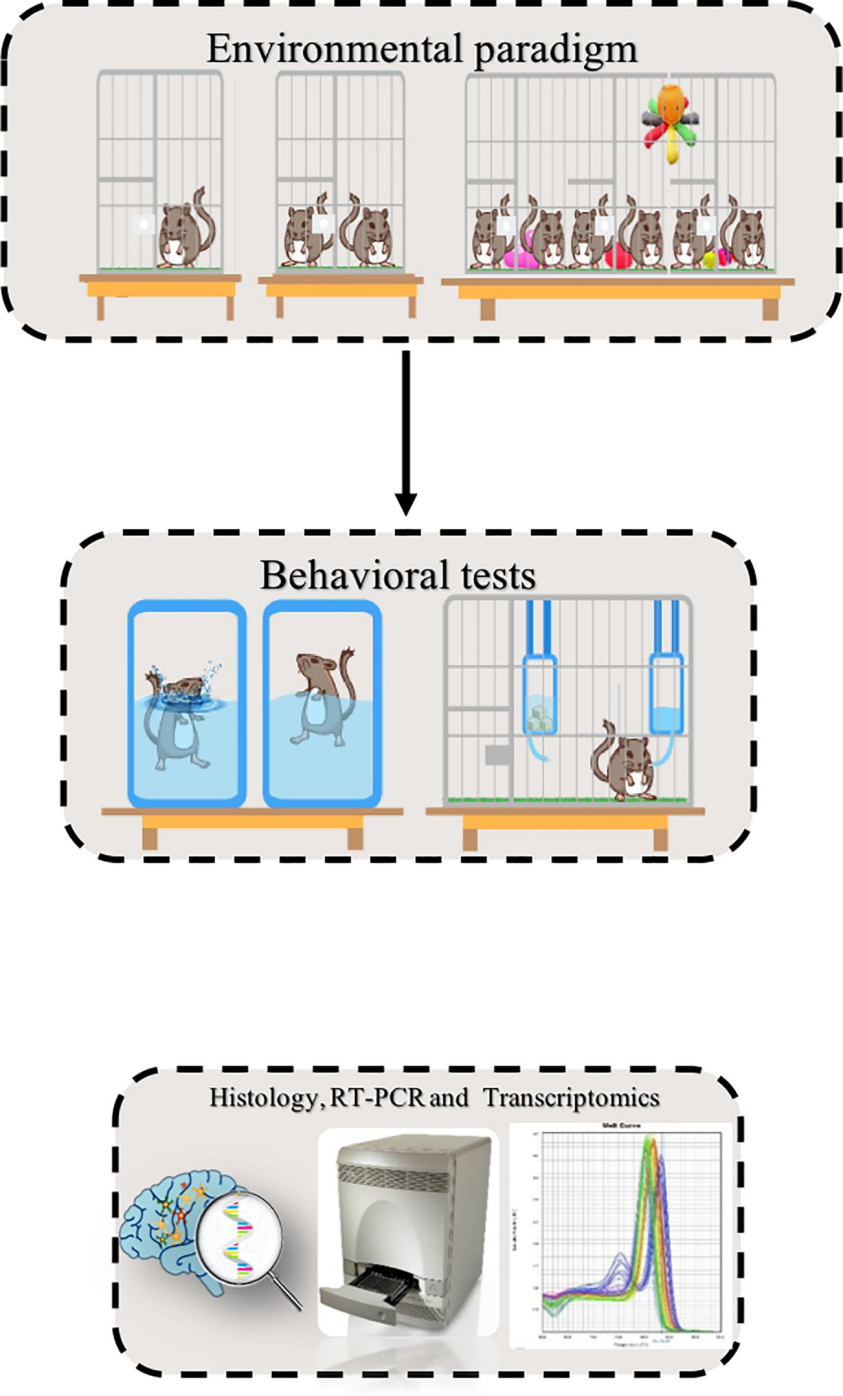
Schematic representation of the study design.

A total of 60 rats were randomly divided into multiple groups (ten rats per each group unless indicated). ***Group I*** included two animals housed per cage (Standard rats). ***Group II*** was isolated rats, one per cage for a period of six weeks (environmentally isolated rats- EI) [16]. ***Group III*** was isolated one per cage for a period of six weeks, and an acute fluoxetine treatment started at the sixth week of the isolation. Fluoxetine (25 mg/kg) was administered daily for seven days [17]. ***Group IV*** was isolated one per cage for a period of six weeks and an acute treatment with etanercept was given for three days in the sixth week. ***Group V*** included isolated rats treated with normal saline (NS). ***Group VI*** included ten rats housed together in a large cage for six weeks in an enriched environment, where various toys were changed three times weekly during the six weeks of experimental housing. The cage dimensions were (1.5 m x 0.5 m x 0.7m), the beading was changed every day, while the toys were changed three times a week. Each time, the toys were removed washed and half of them were taken back to the cage, while the other half was changed. Using a total of 10–8 toys each time [18]. A schematic representation of the study design is depicted in (Fig 1).

We choose two types of drugs an SSRIs (fluoxetine) and an anti-inflammatory (etanercept). Following the five weeks and rats were treated at the 6th week. After that, introduced to the behavior test in the 7th week then sacrificed. The dose of fluoxetine used was 25mg/kg, a fluoxetine syrup was added to the drinking water for seven days. Animals consumption of water was monitored during the isolation period as the oral doses of Fluoxetine were added to the drinking water. Etanercept treated group was treated by 5mg/kg i.p for three days. As a control for the injection we employed normal saline injections (i.p) in EI group.

### Behavioral studies

#### Forced swimming test (FST)

The animals were subjected to FST between 8:00 am to 12:00 pm for two days. On the first day, the animals will be placed into a large cylinder container (30 cm × 45 cm) of 22–24°C water for a 15-min period and then allowed to rest. On the second day, the rats were placed in the same conditions however the test period lasts for five minutes. The activity during the second swim test was video-recorded for subsequent scoring. The swimming behaviors including immobility and mobility were characterized as climbing and diving [19].

#### Sucrose Neophobia (SNP)

The Sucrose Neophobia is used to assess anxiety-like behavioral, neophobia to a novel taste (sucrose) in the first exposure. It was conducted in the home cage from where the water was removed between 8:00 am. And 10:00 am [3]. Meanwhile, 2% sucrose solution was introduced to the rats in the home cage filled in the normal water bottle (Luedtke et al. 2014). Rats were allowed to drink this solution for 30 minutes. Then the amount of consumed water and sucrose are calculated as: sucrose consumption (ml)**/** (sucrose consumption (ml) + water consumption (ml)) × 100% [20].

#### Sucrose Preference (SPT)

Two days after the SNP, the SPT was conducted to assess depression-like behavior and anhedonia. The drinking water was removed between 8:00 am. and 10:00 am. Then, a 2% sucrose solution was introduced to the rats in the home cage filled in the regular water bottle [21]. Rats were allowed to drink this solution for 30 minutes. The amount of sucrose consumption was calculated as previously mentioned in SNP.

### Molecular studies

#### Brain tissue preparation

After the behavioral studies, rats were euthanized using CO2 exposure. The brains were rapidly removed, snap-freezed in liquid nitrogen and stored at −80°C until needed. On the day of the experiment, brains were cut in half longitudinally, and the hippocampal brain region was isolated then washed with normal saline and used for molecular tests and histological examination.

#### Cresyl violet staining

Half brains were kept in 10% formaldehyde solution for two days. Then, they were processed into thin slices and subsequently, exposed to 0.5% cresyl violet staining as described previously [22].

#### Immunofluorescence

A 4% paraformaldehyde fixed brain sections derived from wild-type mice were processed for immunofluorescence staining. Sections were exposed permeabilization, followed by blocking, and primary and Alexa-conjugated secondary antibody staining as previously described [23]. Primary antibodies used were guinea pig anti-NeuN (1:250, Synaptic System, catalog number 266 004), mouse anti-GFAP (1:2500, Novus Biologicals, catalog number NBP1-05197SS), and rabbit anti-TLR7 (1:500, Novus Biologicals, catalog number NBP2-24906SS). We captured the high-resolution confocal image using a Zeiss LSM-510 META confocal microscope, with a C-Apochromat (40x/1.2 W Corr) objective lens. The multi-track acquisition was achieved with excitation lines at 633 nm for A647, 543 nm for Alexa 568, and 488 nm for Alexa 488.

#### Quantification of mRNA Using qRT-PCR

The RNA of hippocampal tissue was isolated and purified was performed using the PureLink^™^ RNA Mini Kit according to the manufacturer’s instructions. After that, the RNA concentration was measured using Nanodrop spectrophotometer (Thermo Scientific, Wilmington, DE, USA). Next, extracted RNA from hippocampus tissue was subjected to reverse transcription and amplified using the High-Capacity cDNA Reverse Transcription Kit according to the manufactures instructions. The synthesized cDNA was then stored at -20°C, until needed. Quantitative real-time polymerase chain reaction (qRT-PCR) analysis was used to determine TLR pathway components: TLR3, TLR4, TLR7, TLR9, MYD88, IRF5, IRAK4, TRAF6. S1 Table represents the primer sequences used in the study. The qRT-PCR was performed using an Applied Biosystems 7500 QPCR detection system, and the analysis was conducted using 7500 software, version 2.0.1 according to the supplier recommendation (Applied Biosystems, Foster City, CA, USA).

#### Transcriptomics in the NAc of enriched and isolated rats

A secondary analysis of data from [24] was conducted to assess TLR signaling in nucleus accumbens of enriched and isolated rats self-administering saline (control) vs. cocaine. The Ingenuity Pathways Analysis (IPA), Canonical Pathways analysis, uses a Fisher’s exact test to identify significantly-regulated gene sets among many well-defined pathways.

### Statistics

The data collected measured in this thesis were expressed as the standard error of the mean (SEM). Differences between the groups were determined using GraphPad Prism 6 software (GraphPad Software, San Diego, CA, USA). For short-term isolation studies, we used a student t-test. For analyzing body weight tracking, we used two-way ANOVA followed by Bonferroni's multiple comparison tests. For the rest of analyses, we used one-way analysis of variance (ANOVA) followed by Tukey's multiple comparisons test. The statistical differences were considered significant at P<0.05

## 3. Results

**Fig 2:**
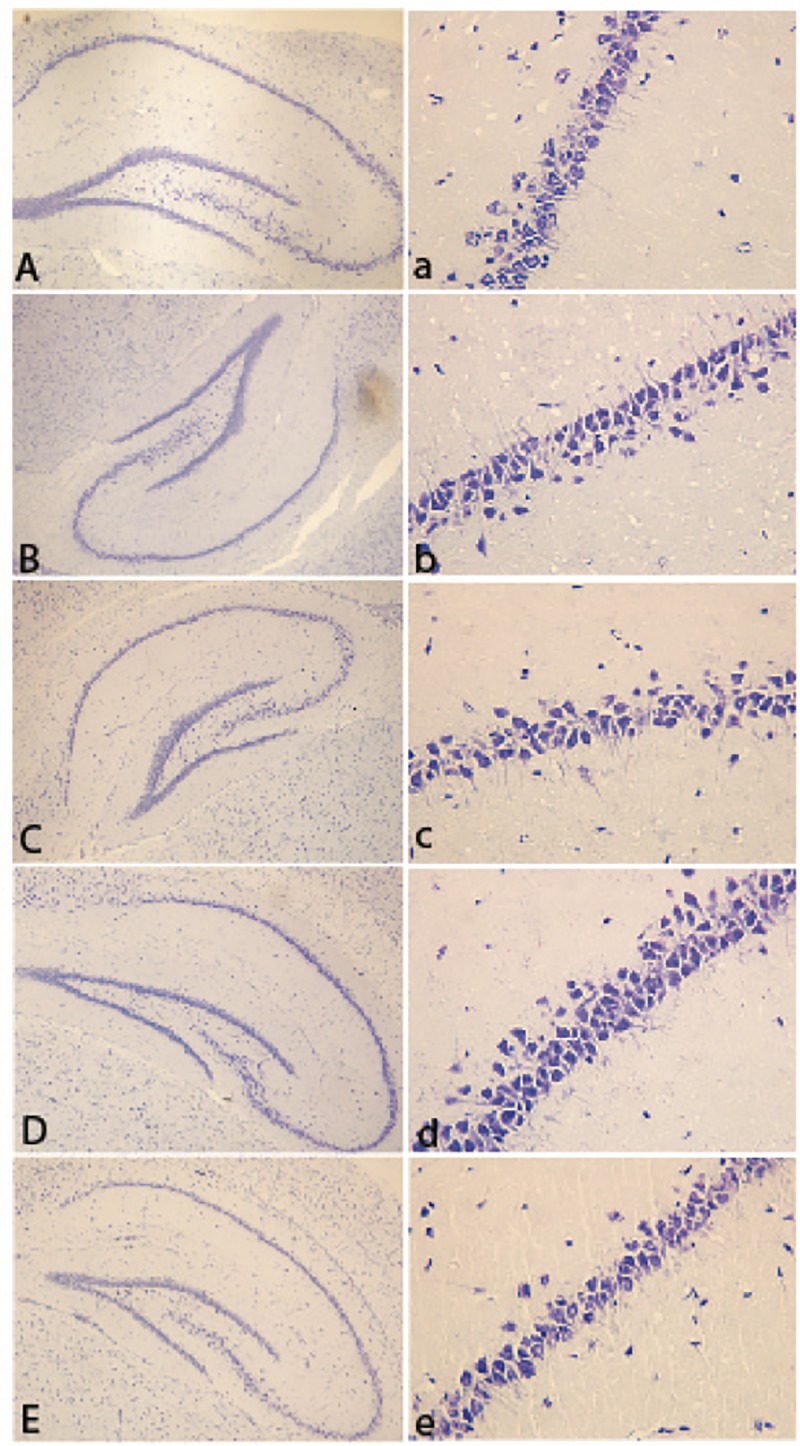
Histological examination of the hippocampal region using Cresyl violet staining. The effects of environmental paradigm and pharmacological intervention on hippocampal neurons were measured using Cresyl violet staining. Representative micrographs of the CA1 and the hippocampus. The first column of micrographs was captured at Low magnification under a light microscope; the remaining micrographs were captured at higher magnification under a light microscope. (A, a) representative images of the hippocampus region in the control group (B, b) isolated, (C, c) flux treated, (D, d) etanercept treated, and (E, e) the EE condition.

To examine the gross morphology of the groups, we conducted cresyl violet staining using sagittal brain sections. We did not observe any morphological alterations in the hippocampus between the groups (Fig 2).

### The effect of an environmental paradigm in SPT and FST

**Fig 3:**
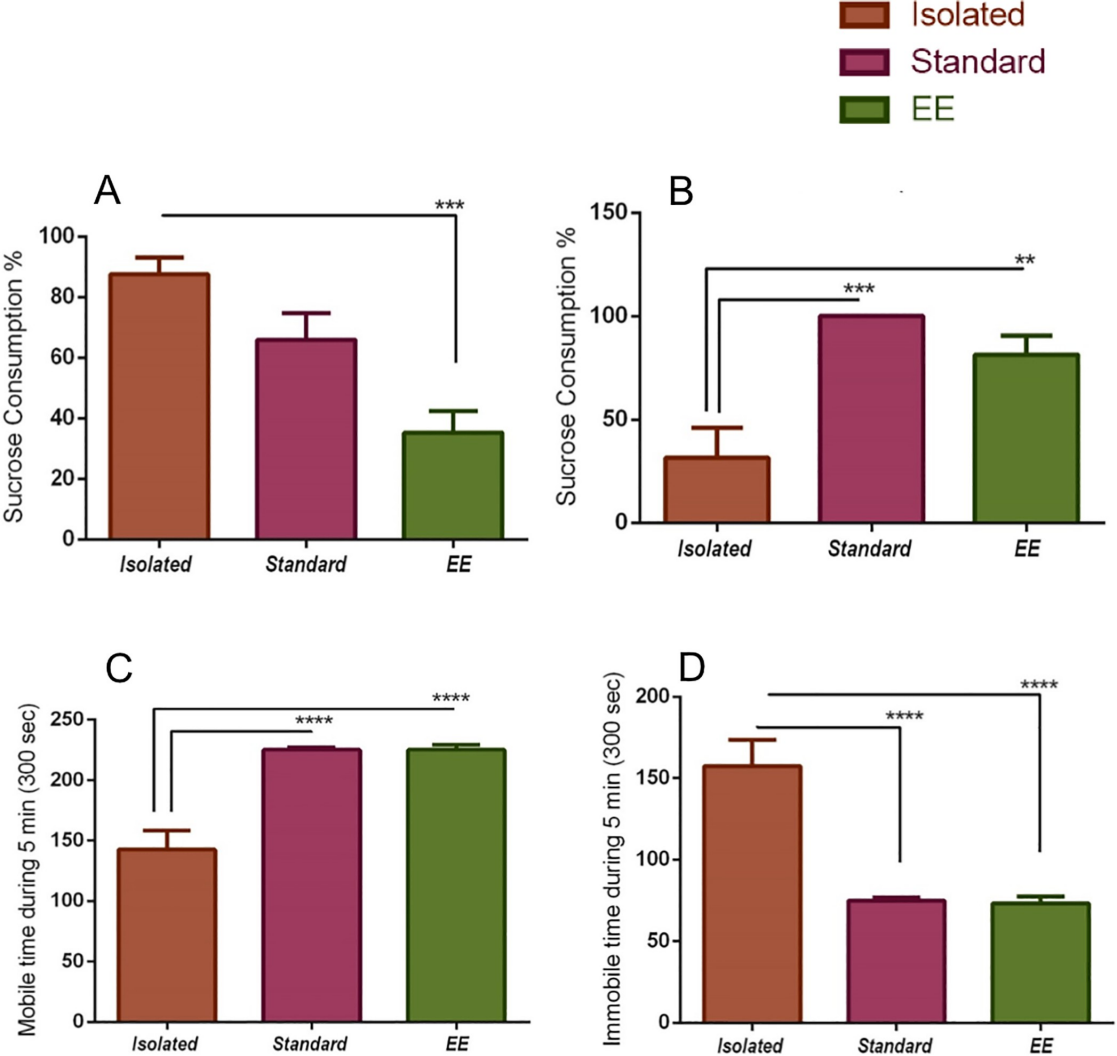
The Effect of environmental paradigm in SPT and FST. Rats were initially exposed to the sucrose to assess neophobia, (n = 6), (A) First-day exposure. On The second day animal was exposed to the sucrose to test depressive-like behavior, (B) Second Exposure. The presence or absence of mobility or immobility behavior were registered every 5s (bins) in the second session of FST, (C) showed the significance of mobility between paired and. EE to the isolation, (D) shows the significant of immobility between isolation to the paired and EE. n = 6 per group. Results represent the mean±SEM. Data were analyzed using one-way ANOVA followed by Tukey's test. **P<0.01, ***P<0.001 ****P<0.0001.

In these experiments, three animal housing groups were used: isolated, standard, and enriched. Upon first sucrose exposure, the isolated group consumed more sucrose than the other groups. This consumption was significant compared to the EE group [87.50±5.59 ml vs. 35.31±7.13 ml (P = 0.0004)] (Fig 3A). The second sucrose exposure showed that the isolated group consumed less sucrose than the standard group [31.74±14.30 ml vs. 100±0 ml (P = 0.0005)] and the EE group [31.74±14.30 vs. 81.42±9.11 ml (P = 0.007)] (Fig 3B).

FST measures of despair and loss of hope were taken during a five-minute test session wherein time of mobility and immobility were analyzed. The results showed that mobility was higher in EE [225.0±4.281 Sec vs. 142.5±15.905 Sec (P<0.0001)] and standard groups [225.0±1.825 Sec vs. 142.5±15.905 Sec (P<0.0001)] compared to the SI group (Fig 3A). The analysis of immobility indicated that the SI group was immobile compared to the standard group [157.5±15.903 Sec vs. 75.0±1.825 Sec (P<0.0001)] (Fig 3B) and exhibited a despair behaviour. In addition, the SI rats were also substantially immobile compared to the EE group [157.5±15.903 Sec vs. 73.33±4.216 Sec (P<0.0001)] (Fig 3B).

### The effect of pharmacological intervention in SPT and FST

**Fig 4:**
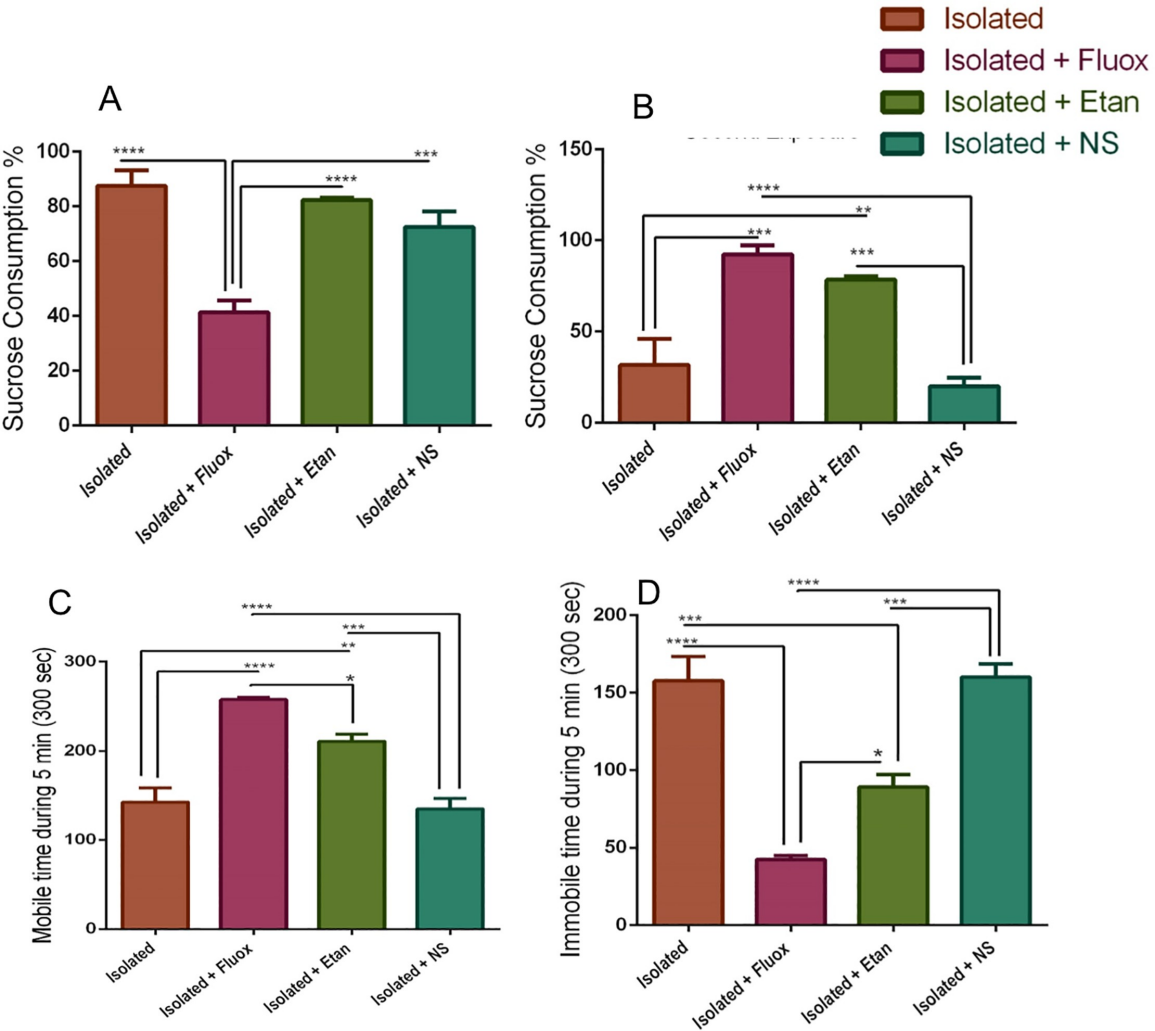
The Effect of pharmacological intervention in SPT and FST. Rats were initially exposed to the sucrose to assess the neophobia (A) First-day exposure. On the second day rats were exposed to the sucrose to test depressive-like behavior, (B) Second exposure. The presence or absence of mobility or immobility behavior were registered every 5s (bins) in the second session of FST, n = 6 per group. (C) Showed the significant of mobility between fluoxetine treatment and etanercept to the isolation, (D) shows the significant of immobility between isolation to fluoxetine treatment and etanercept. Results represent the mean±SEM. Data were analyzed using one-way ANOVA followed by Tukey's test. *P<0.05, **P<0.01, ***P<0.0001.

After confirming the EI approach as a model of depression, the functional effects of fluoxetine and etanercept were analyzed. The results showed that sucrose consumption in the etanercept treated group was comparable to the isolated group (Fig 4A). However, fluoxetine-treated rats consumed a smaller amount of sucrose than the isolated group [41.388±4.312 ml vs. 87.5±5.590 ml (P = 0.0004)] and the etanercept and NS groups [41.38 ±4.43-fold vs. 82.33±0.80-fold (P<0.0001)], [41.38 ±4.43-fold vs. 72.5 ±5.7-fold (P = 0.0005)] (Fig 4A).

On the second sucrose exposure, anhedonia examination studies showed that fluoxetine treatment increased sucrose consumption compared to the isolated group [92.333±4.8 ml vs. 31.743±14.308 ml (P = 0.0002)] (Fig 4B), indicating the remission of depressive-like behaviour. Notably, etanercept acute treatment resulted in reduced depressive-like behaviour, which was demonstrated by a significant increase in sucrose consumption compared to the isolated rats [78.46±1.696 ml vs. 31.743±14.308 ml (P = 0.002)] (Fig 4B). The fluoxetine-treated group consumed more sucrose than the NS- treated group [92.33 ±4.8 -fold vs. 20.08±4.5-fold (P<0.0001)], while the etanercept-treated group consumed more sucrose than the NS- treated group [78.46±1.7-fold vs. 20.08 ±4.5-fold (P = 0.0002)].

The antidepressant effect of fluoxetine and etanercept treatments were then examined using FST. The results showed that fluoxetine treatment increased mobility time compared to the isolation group [257.5±2.5 Sec vs. 142.5±15.903 Sec (P<0.0001)] (Fig 4C) and reduced immobility time compared to the same group [42.5±2.5 Sec vs. 157.5±15.9 Sec (P<0.0001)] (Fig 4D). The acute etanercept treatment increased mobility time compared to the isolated group [210.833±8.001 Sec vs. 142.5±15.903 Sec (P = 0.001)] (Fig 4C) and reduced immobility time compared to the same group [89.1667±8.001 Sec vs. 157.5±15.903 Sec (P = 0.0005)] (Fig 4D). However, the reduction in despair among etanercept-treated rats was less than in the fluoxetine-treated group [89.1667±8.001 vs. 42.5±2.5 Sec (P = 0.0)] (Fig 4D). The mobility time of the NS-treated group compared to fluoxetine and etanercept treated groups was [135±11.7 Sec vs. 257.5±2.5 Sec (P<0.0001)], [135±11.7 Sec vs. 210.833±8.001 Sec (P = 0.0004)] (4C). The immobility time of the NS group compared to fluoxetine and etanercept groups was [160 ±8.65 Sec vs. 42.5±2.5 Sec (P<0.0001)], [160 ±8.65 Sec vs 89.16 Sec (P = 0.0003)] (4D).

### TLR7 and its signaling components mRNA expression in the hippocampus

**Fig 5:**
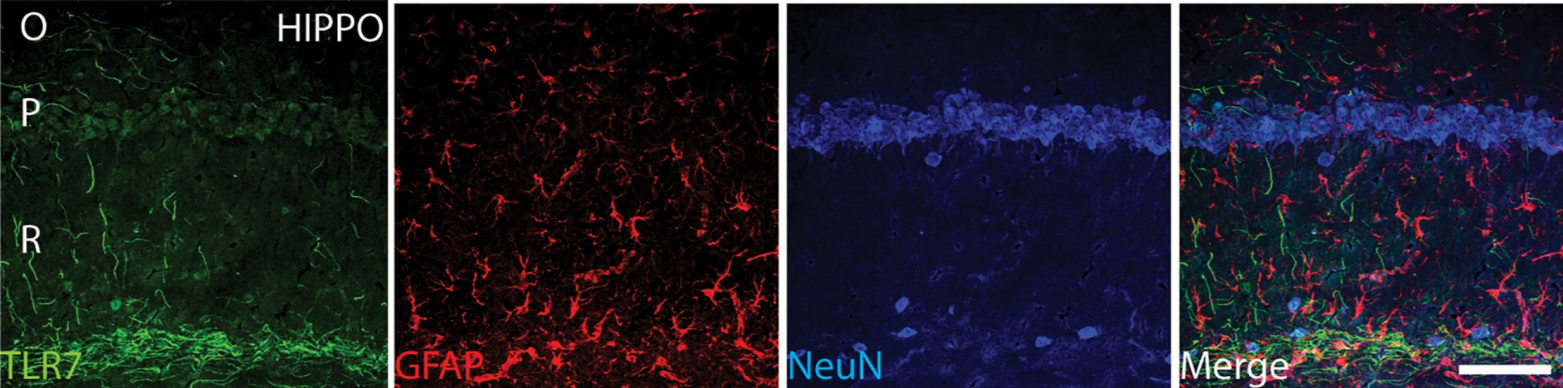
The expression of TLR-7 in the hippocampal brain region. The immunofluorescence of TLR7 recognized by Alexa 488, green. GFAP recognized by Alexa 594, red. NEUN recognized by Alexa 633, (blue) and merged image in the hippocampal region. NeuN and GFAP were applied to show the distribution of TLR7 within neuronal and supportive tissue populations. Scale bar 80 μm.

**Fig 6:**
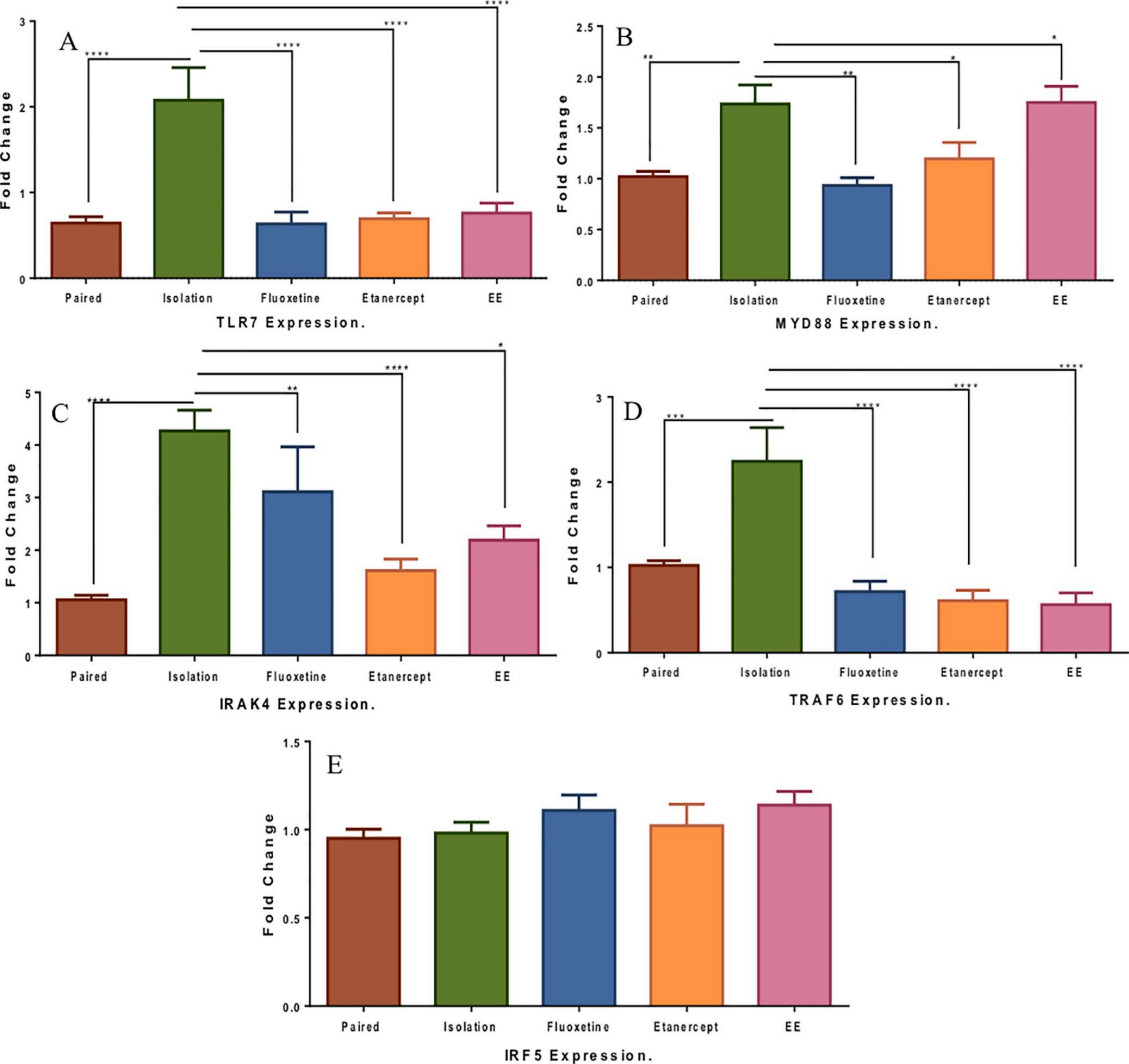
TLR7 and its signaling components mRNA expression in the hippocampus. The mRNA analysis of TLR7 and its signaling pathway in the experimental groups. The mRNA expression level for TLR7 pathway hippocampus brain region determined by RT-PCR analysis. (A) TLR7. (B) MYD88. (C) IRAK4. (D) TRAF6. (E) IRF5. Using GAPDH as an internal control. Data are expressed as fold change. Data normalized to the paired (control nondepressed group). The results are presented as the mean±SEM from two independent experiments with triplicate (n = 3 animals per group). Data were analyzed using. One-way ANOVA followed by Tukey's test. *P<0.05; **P<0.01; ***P<0.001; ****P<0.0001.

Following the behavioral studies, we aimed to link the functional studies to the molecular level. We posited that an alteration drives the behavioral phenotypes observed in the environmental paradigm studies are related to the TLR7 pathway. We examined the expression of TLR7 within the brain hippocampal region, and our immunofluorescence study indicated that TLR7 is expressed within the hippocampus (Fig 5). To validate this hypothesis, the level of expression of TLR7 and its pathway components, including MYD88, IRAK4, TRAF6, and IFR5, were measured. TLR7 expression level increased compared to the standard group due to prolonged isolated housing [2.24 ±0.38-fold vs. 0.89 ±0.1-fold (P<0.0001)]. Moreover, the mRNA level was reduced in the isolated group treated with fluoxetine compared to the isolated group [0.63 ±0.13-fold vs. 2.24 ±0.38-fold (P<0.0001)]. Similarly, the etanercept treatment decreased TLR7 compared to the isolated group [0.69 ±0.06-fold vs. 2.07 ±0.38-fold (P<0.0001)]. Conversely, EE housing resulted in a reduction of TLR7 expression compared to the isolated group [0.76 ±0.11-fold vs. 2.07±0.38-fold (P<0.0001)]. There were also no notable differences in comparison to the standard, fluoxetine, and etanercept groups (Fig 6A). The isolated group, however, differed from the group housed in standard conditions and treated with fluoxetine [2.24 ±0.38-fold vs. 1.03±0.08-fold (P<0.0001)]. Additionally, the analysis showed a difference in the isolated group compared to the NS treated group [2.24 ±0.38-fold vs. 0.83 ±0.14-fold (P<0.0001)].

The MYD88 level was also analyzed. MYD88 bridges TLRs to the downstream signaling elements. We found that MYD88 increased in the chronically isolated group compared to the standard group [1.73±0.18-fold vs. 1.05 ±0.05-fold (P = 0.01)]. Furthermore, MYD88 expression was substantially reduced after treatment with fluoxetine compared to the isolated group [0.93 ±0.07-fold vs. 1.73±0.18-fold (P = 0.001)] and etanercept treatment reduced MYD88 compared to the isolated group [1.19 ±0.16-fold vs. 1.73±0.18-fold (P = 0.04)]. However, EE housing resulted in a nonsignificant increase in MYD88 expression compared to the isolated group and the fluoxetine-treated group [1.75±0.15-fold vs. 0.93 ±0.07-fold (P<0.01)] (Fig 6B). Comparison of the isolated group to the standard fluoxetine group [1.73 ±0.18-fold vs. 1.02 ±0.06-fold (P = 0.04)] suggests that MYD88 is affected by EI, EE, and pharmacological intervention. These results imply that MYD88 could be a potential target for treating depression.

Correspondingly, IRAK4 expression increased after isolation compared to the standard housing group [4.26±0.39-fold vs. 1.05±0.08-fold (P<0.0001)] and decreased after treatment with etanercept [4.26±0.39-fold vs. 1.61±0.22-fold (P<0.0001)], while EE reduced IRAK4 compared to the isolation group [2.18±0.27-fold vs. 4.26±0.39-fold (P<0.0001)]. Conversely, fluoxetine treatment yielded a significant reduction compared to the isolation group [1.26±0.5-fold vs. 4.26±0.39-fold (P<0.0001)] (Fig 6C). The fold change of IRAK4 expression for the isolation group compared to the NS group were [4.26 ±0.39-fold vs. 0.69 ±0.07-fold (P<0.0001)] and for the isolation group compared to the standard fluoxetine group were [4.26 ±0.39-fold vs. 1.01 ±0.06-fold (P<0.0001)].

To further investigate the TLR pathway, the mRNA level of TRAF6 was examined. TRAF6 increased in the isolated group compared to the standard group [2.24±0.39-fold vs. 1.02±0.05-fold (P<0.0001)]. Conversely, the TRAF6 mRNA level was reduced after treatment with both fluoxetine [2.24±0.39-fold vs. 0.71 ±0.12-fold (P<0. 0001)] and etanercept [2.24±0.39-fold vs. 0.61 ±0.12-fold (P<0. 0001)], demonstrating an effect of treatment on TRAF6 mRNA expression level. The EE housing resulted in a reduction in TRAF6 mRNA level compared to the isolated group [0.56±0.13-fold vs. 2.24±0.39-fold (P<0.0001)]. Furthermore, TRAF6 mRNA in EE housing was comparable to the standard group, fluoxetine-treated group, and the etanercept treated group (Fig 6D). The isolated group differed from the NS-treated group [2.24 ±0.39-fold vs. 0.69 ±0.07-fold (P<0.0001)] and the standard group treated with fluoxetine [2.24 ±0.39-fold vs. 0.71 ±0.12-fold (P = 0.0007)]. Finally, the IRF5 mRNA expression analysis in the hippocampus showed there was no change among the groups (Fig 6E).

### The qPCR expression measurements of TLR3,4 and 9 in the experimental groups

**Fig 7:**
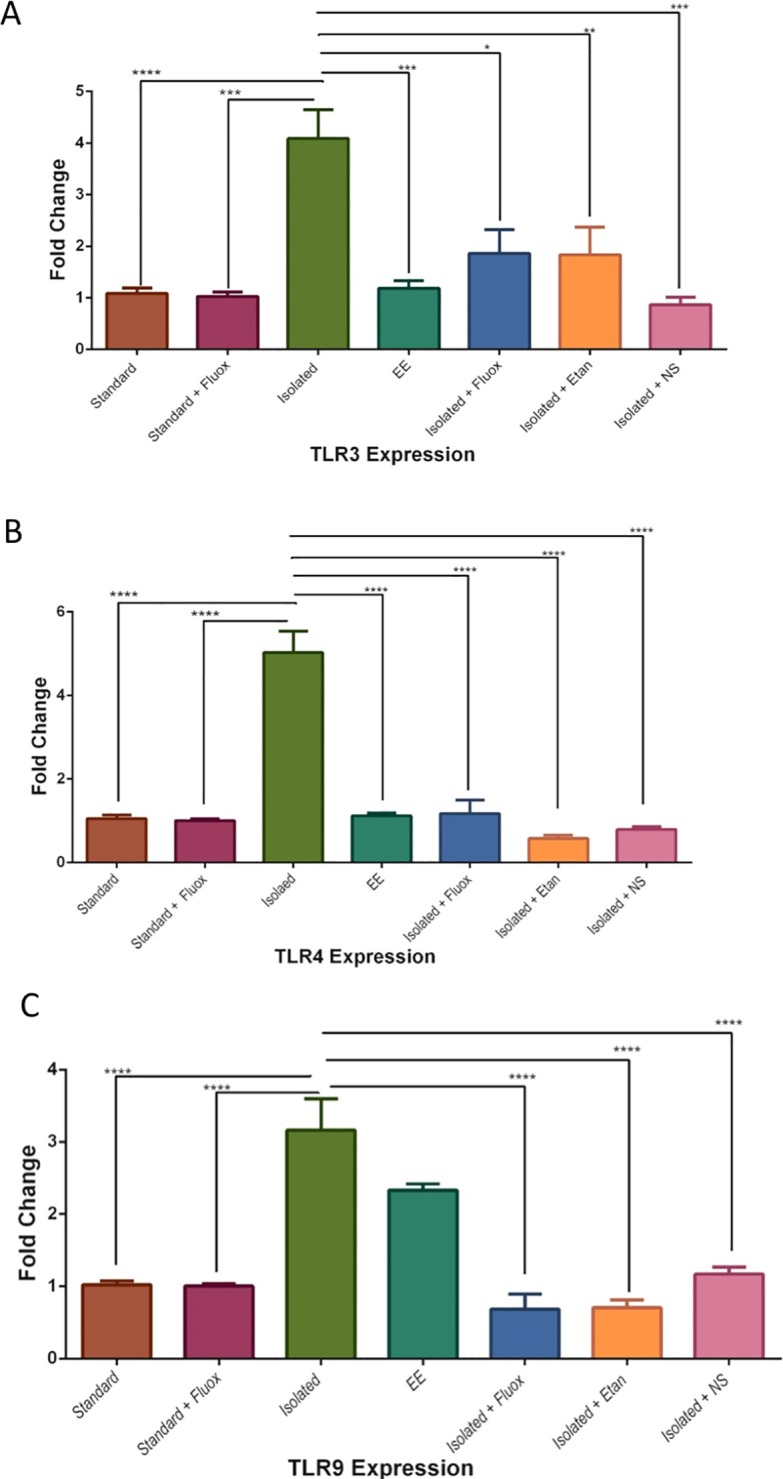
The molecular examination of TLR 3, 4 and 9 in the experimental groups. The molecular examination of TLR3,4 and 9 in the experimental groups. The mRNA expression level for TLR3. TLR4 and TLR9 in hippocampus brain region determined by RT-PCR analysis with GAPDH as an internal control. Data expressed as fold change. Data normalized to the paired (control nondepressed group). The results are presented as the mean±SEM from two independent experiments with triplicate (n = 3 animals per group). Data were analyzed using. One-way ANOVA followed by Tukey's test. *P<0.05; **P<0.01; ****P<0.0001.

EI resulted in an increase in TLR3 mRNA level in the hippocampus compared to the standard group [4.08±0.55-fold vs. 1.08±0.10-fold (P<0.0001), whereas the mRNA level of TLR3 was reduced in the isolated group treated with fluoxetine [4.08±0.55-fold vs. 1.86±0.45 -fold (P = 0.01)]. Similar findings were observed in the isolated group compared to the isolated group treated with etanercept [4.08±0.55-fold vs. 1.86 ±0.53-fold (P0.001)]. However, EE housing results in a reduction in TLR3 mRNA expression compared to the isolated group [1.18±0.14-fold vs. 4.08±0.55-fold (P = 0.0008)] but lacks significance compared to the standard, fluoxetine and etanercept-treated groups, suggesting that environmental conditions exert strong effects compared to the pharmacologically treated groups (Fig 7A). Isolation compared to NS was [4.08 ±0.55-fold vs. 0.83 ±0.1-fold (P = 0.001)], while isolation compared to standard fluoxetine was [4.08 ±0.55-fold vs. 1.02 ±0.08-fold (P = 0.0003)].

TLR4 was also examined and the results showed that the expression level of TLR4 increased due to prolonged isolation compared to standard housing [5.02±0.51-fold vs. 1.05 ±0.08-fold (P<0.0001)]. The expression level was, however, reduced after treatment with fluoxetine compared to the isolated group [1.17 ±0.32 -fold vs. 5.02±0.51-fold (P<0.0001)]. Similarly, etanercept treatment resulted in down-regulation of TLR4 expression compared to the isolated group [0.57 ±0.08-fold vs. 5.02±0.51 -fold (P<0.0001(]. Additionally, EE housing resulted in a substantial reduction in TLR4 mRNA level compared to the isolated group [1.11±0.07-fold vs. 5.02±0.51-fold (P<0.0001)]. However, the TLR4 expression level was comparable in the EE housing, standard, fluoxetine, and etanercept-treated groups (Fig 7B). EI compared to NS-treated group was [5.02 ±0.51-fold vs. 0.79 ±0.06-fold (P<0.0001)] while the fold change in the expression level in the EI group compared to standard housed fluoxetine treated group was [5.02±0.51-fold vs. 1 ±0.03-fold (P<0.0001)].

TLR9 was the last member of the TLR family to be evaluated. The mRNA level increased considerably in the chronically isolated group compared to the standard group [3.16 ±0.43-fold vs. 1.02 ±0.04-fold (P<0.0001)]. Additionally, treating the EI group with fluoxetine reduced the TLR9 level compared to the isolated group [0.68 ±0.21-fold vs. 3.16 ±0.43-fold (P<0.0001)]. Similar findings were observed for the etanercept-treated group [0.70 ±0.10-fold vs. 3.16 ±0.43 -fold (P<0.0001)]. This shows that TLR9 expression is affected by pharmacological treatment. Furthermore, the TLR9 level was higher in the EE group than the standard sample group [2.32 ±0.08-fold vs. 1.021 ±0.04-fold (P<0.0002 (] (Fig 7C). These analyses indicate that stressful events triggered the TLRs and their expression is sensitive to pharmacological intervention as well as EE conditions. The analysis in EI compared to NS-treated group was [3.16 ±0.43-fold vs. 1.16 ±0.09-fold (P<0.0001)] while in EI compared to standard housed fluoxetine treated group was [3.16 ±0.43-fold vs. 1 ±0.03-fold (P<0.0001)].

### The transcriptomics studies in the nucleus accumbens

**Fig 8:**
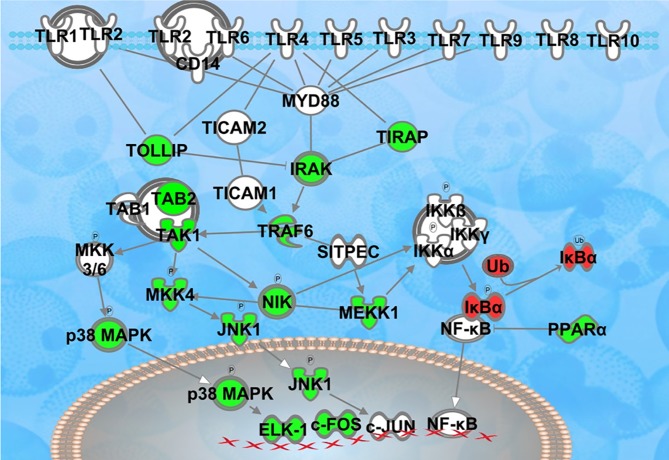
Regulation of TLR-related transcripts in the nucleus accumbens of enriched vs. isolated rats. The figure shows transcripts significantly downregulated (green symbols) or upregulated (red) in the Toll-like Receptor Signaling pathway, as described by Ingenuity Pathways Analysis software. The pathway as a whole was significantly regulated (-log (p) = 1.63).

The nucleus accumbens (NAc) is functionally implicated in the reward system and has been intensively studied in the context of depression [25]. Alterations in this brain region are dependent on hippocampal integrity [26]. We therefore examined the TLR signaling pathway in enriched versus isolated rats. The Toll-Like Receptor Signaling pathway was statistically significant (-log (p) = 1.63) between the two groups. Therefore, more transcripts in this pathway were regulated by enrichment than would be expected by chance. Additionally, the z-score was significant (-2.714), suggesting a directional change with enriched rats showing lower levels of transcripts for the components of this pathway (Fig 8).

## 4. Discussion

We examined the role of TLR7 pathway activation in the hippocampus and the NAc in a model of depression. Multiple members of the TLRs family were elevated in the EI group. This suggests that TLR pathways could be involved molecularly in the pathogenesis of depression. Furthermore, our results indicate that TLR signaling is affected by acute treatment with an anti-inflammatory drug when fluoxetine is used as a positive control for antidepressant effects. We also found that EE housing exhibits a substantial effect on the TLR family suggesting potential involvement of this signaling in multiple brain disorders.

### The effect of environmental paradigm in SPT and FST

To validate our animal model prior to examining the potential antidepressant effects of fluoxetine and etanercept on depressed and anxiety-like behaviours at the molecular level, we began by investigating the environmental paradigm. Our functional assessment was based on studying standard parameters such as 1) anxiety in response to a novel taste (neophobia), 2) lack of pleasure (anhedonia), and 3) hopelessness (immobility time). Anxiety-like behaviour was evaluated using the sucrose preference test, which can determine both pleasure-seeking behaviour and anxiety-like behaviour[20, 27]. A SI approach was employed to induce depression-like behaviour. The results showed that six-weeks of SI increased sucrose consumption in the first exposure of SPT, which suggests SI-induced neophobia, and decreased in the second exposure compared to the control. The significant reduction in sucrose consumption indicates that isolated groups exhibit anhedonia. EE resulted in less sucrose consumption on the first exposure compared to the control, suggesting an enriched paradigm conferred a resilience to anxiety-like behaviour. It then increased in the second session, which means EE prevents the anhedonia. Despair and hopelessness symptoms were assessed using FST, a standard measurement of depressive-like behaviour in rodents. FST measured despair and loss of hope during the five-minute test session wherein mobility and immobility times were analysed. SI was highly immobile compared to the standard group while EE was highly mobile. In line with these findings, previous studies have shown that EI reduces sucrose consumption and increase immobility time while performing FST [28].

### The effect of pharmacological intervention in SPT and FST

We then examined the behavioral effects of fluoxetine and etanercept as antidepressants. Treatment with etanercept produced a reduction in anxiety-related and depressive-like behaviour in rats [29, 30]. Moreover, anti-cytokine drugs have been shown to reduce depression and improve symptoms in humans by regulating mediators of inflammation [8]. In a previous study, chronic administration of etanercept at a dose of 0.8 mg per kg for eight weeks reduced anxiety-like behaviour compared to control rats by increasing time spent in the open arms and the number of open arm entries in the elevated plus maze. It also reduced immobility time in the FST task [29] indicating its antidepressant effects. However, the effectiveness of etanercept as an anxiolytic and antidepressant agent at the molecular level has yet to be investigated.

After validating the EI approach as a model of depression and anxiety, we analysed the functional effects of fluoxetine and etanercept. The fluoxetine-treated group showed decreased sucrose consumption in the first exposure, suggesting that acute fluoxetine treatment affects anxiety-like behaviour in the isolated group. On the second sucrose exposure, the anhedonia studies showed that fluoxetine treatment increased sucrose consumption compared to the isolated group. Therefore, compared to the isolated group, fluoxetine reduces the anhedonia effect. In line with these findings, a previous study found that chronic administration of fluoxetine increased sucrose consumption and reversed anhedonic feelings in a genetic model of depression. Fluoxetine also reversed depressive-like behavior and exerted an anxiogenic effect [31].

Conversely, sucrose consumption in the isolated group treated with etanercept was comparable to the isolated group, suggesting that acute etanercept treatment lacks significant neophobia effects. It also indicates that etanercept may not affect anxiety-like behaviour. Etanercept increased sucrose consumption in the second session and, compared to the isolated group, reduced the anhedonia effect. Similarly, treating a lipopolysaccharide model of depression with minocycline, a tetracycline antibiotic, has been shown to be effective in reversing anhedonic feelings. It also reversed poor sociability, weight loss, and a loss of appetite. Moreover, at the molecular level, it reversed the elevation in the inflammatory markers [32].

When the effect of treatments on FST were assessed, both fluoxetine and etanercept increased mobility time compared to the SI. This test is usually employed to evaluate the effectiveness of antidepressant therapy in rodents. However, the reduction in despair in etanercept-treated rats was less than in the fluoxetine-treated group. Similar findings were observed in fluoxetine treated rodents when performing FST tasks [33]. This suggests that the effectiveness of etanercept, although significant, was not as great as fluoxetine. The chronic administration of etanercept has been shown to be beneficial in reversing depressive-like behaviour. For instance, the eight-week treatment was effective in reducing the despair measured by FST. Furthermore, it was effective in lowering anxiety-like behaviour measured by the elevated plus maze. These results suggest that introducing anti-tumor necrosis factor agents to the antidepressant regimen would be a constructive treatment strategy [29]. Consistent findings regarding etanercept treatment and improvement in despair measured by FST was also reported in restrained rats [30].

Notably, a study published in the Lancet has concluded that treatment with etanercept in humans is linked to the alleviation of depression symptoms [34], even though etanercept does not cross the BBB [35]. Previous studies have shown that the peripheral administration of etanercept is effective in managing neuroinflammation [36], modulating adult neurogenesis, and in corticosterone-induced synaptic alterations [37], this effect could be due to a reduction in the circulating inflammatory markers. A previous study reported that the administration of etanercept resulted in a reduction in neuroinflammation in a brain injury rat model. These improvements could be directly mediated by modulating the hepatic response during the acute stage of brain injury, which changes the level of chemokine and neutrophil in the circuitry [38]. Another study reported that peripheral administration of etanercept leads to a reduction in the circulatory TNF-α, which indirectly affect the level of TNF-α in the CNS [39]. Thus, it is speculated that etanercept exerts its central effects via direct modulation of the peripheral level of TNF-α [36]. TNF-α in the periphery has functional effects on microglia, as it stimulates the activity of microglial cells leading to increased production of TNF-α by microglia [40, 41]. Etanercept therefore has excellent potential as an adjunctive therapy in major depressive disorder, which can be usefully explored in future studies.

### TLR7 and its signaling components mRNA expression in the hippocampus

Next, we posited that the behavioral and molecular phenotypes observed in our study using the environmental paradigm are driven by an alteration in the TLR pathway, especially TLR7. To validate this hypothesis and evaluate the expression of TLRs and their pathway components, we investigated the expression of TLR3, TLR4, TLR7, and TLR9, and their pathway components, including MYD88, IRAK4 TRAF6 and IFR5, after stressful events caused by SI. We began our investigation by examining TLR7.

TLR7 can be triggered by single-stranded RNA, leading to death in neuronal populations, a substantial increase in the inflammatory mediators, and neuronal toxicity [42]. Compared to standard housing, the expression level increased due to the isolation. Indicating that TLR7 expression is affected by environmental conditions, and treatment with fluoxetine and etanercept can reduce the elevation in TLR7 expression. However, EE housing results in a reduction of TLR7 compared to the ES and is comparable to the standard, fluoxetine, and etanercept groups. A recent study has shown that TLR7 transcript level is elevated in a genetically modified depressive model [3]. We then examined other components of the TLR pathway. For instance, we evaluated the mRNA expression level of MYD88, an adapter protein that plays a vital role in innate and adaptive immunity and bridges TLRs to the downstream signaling elements [43]. MYD88 increased due to isolation and reduced after treatment with fluoxetine and etanercept. EE housing, however, induced MYD88 expression, suggesting that MYD88 is affected by EI, EE, and pharmacological intervention. These results implied that MYD88 could be a potential target for treating depression. In line with this evidence, a postmortem study has shown that MyD88 was elevated in the prefrontal cortex of schizophrenic and depressed patients [15]. We also examined IRAK4, which was induced by isolation and then reduced by acute treatment with either fluoxetine or etanercept. We then evaluated TRAF6, where the mRNA level increased in the isolation group and decreased following treatment with fluoxetine and etanercept. Finally, we examined the level of expression of IRF5, which is a member of the interferon regulatory factor family and an essential modulator of TLR signaling. This did not change after isolation, EE, and treatments.

Our studies have shown that a TNF-α inhibitor is effective in reducing inflammatory markers, and that could be the mechanism underlying the antidepressant effects. One the other hand, fluoxetine has shown a significant impact on reducing these inflammatory markers. The mechanism underlying this effect on the expression of TLR is unknown. Yet, two possible mechanisms may underly the generalized reduction in TLR and other inflammatory markers. One of them is that increased level of serotonergic signals on immune cells modulates the synthesis of inflammatory markers. Another possible mechanism is that antidepressant agents enhance the synthesis of cyclic adenosine monophosphate, which in turn reduce the level of inflammatory cytokines[5, 44].

### The molecular examination of TLR3, 4 and 9 in the experimental groups

Other members of the TLR family were also examined. In general, the expression of a TLR is induced by several protocols of psychological stress in rodents such as CMS and social disruption. In the context of neurobiology, the members of the TLR family most studied are TLR-4 and TLR-9, the latter of which has been presented as a specific regulator of the adrenal response to inflammatory stimuli [4, 15, 45–48]. Alterations in TLR signaling were reported in mouse models of brain disorders [49]. Here we found that isolation resulted in an increase in TLR3 mRNA level and was reduced after treatment by both fluoxetine and etanercept, suggesting that both antidepressant and anti-inflammatory treatments influenced the mRNA expression level of TLR3. Furthermore, EE housing does not have a significant impact on TLR3 compared to the standard, fluoxetine, and etanercept-treated groups, suggesting that EE housing exerted strong effects compared to the pharmacologically treated group. The mRNA expression of TLR3 and TLR4 has been found to increase in the prefrontal cortical regions of individuals with depression that died by suicide and depressed non-suicide subjects [7]. TLR3-deficient mice have been shown to exhibit a reduced anxiety-like behaviour implying potential roles for TLR3 signaling in anxiety disorders [50]. Moreover, TLR4 increased in CMS and social disruption animal studies [15]. Our results indicated that TLR4 was involved in the regulation of stress-induced neuroinflammatory signals. Compared to standard housing, the expression level of TLR4 increased as a result of isolation. In vitro studies suggest that using tricyclic antidepressants strongly inhibits TLR2 and 4 signaling [51]. In human postmortem studies of psychiatric disorders, TLR4 has been found to have been altered [10]. Finally, we evaluated TLR9. The mRNA level increased considerably in the isolation group compared to the standard group and reduced after treatment with both fluoxetine and etanercept. It has recently been shown that TLR9 deficiency protects against lymphocyte apoptosis induced by chronic stress. TLR9 deficiency was also found to reverse the elevation of plasma IL-1β, IL-10 and IL-17 levels and a decrease in plasma IFN-γ level under conditions of chronic stress [52]. Postmortem analysis[53], as well as the peripheral level in the circuitry of depressed patients, showed that TLR3, TLR4, TLR5, TLR7, TLR8, and TLR9 all increased. In their study, they found that the level of mRNA expression of multiple members of the TLR family was significantly reduced in the periphery after one month of treatment with antidepressants. In fact, some of them (TLR3, TLR4, TLR5, and TLR7) were normalized. On the other hand, TLR1, TLR2, and TLR6 mRNA levels were lower than healthy individuals. This study supports our findings regarding the interaction between antidepressant treatment and the expression level of TLR [51]. Whether this is due to an elevation in the circuitry that is leaked to the brain, or it originates in the brain due to neuronal insults triggered by stressful life events, is for future studies to determine.

A previous study has reported that in lipopolysaccharide treated rats, a pharmacological animal model exhibiting depressive-like symptoms, both desipramine and fluoxetine prevented TNF-α release [54]. Another research has shown that chronic fluoxetine treatment in CMS animal model exhibits significant central and peripheral modulation of inflammatory cytokines. Indicating that chronic administration of fluoxetine exhibit therapeutic effects by modulating of IL-1β[55].

Attempts to enhance the treatment of depression by exploring inflammatory mechanisms and employing anti-inflammatory agents hold great potential. Although the mechanism underlying the effectiveness of etanercept in reducing depression symptoms has not been tested mechanistically, it may be useful as an adjuvant agent with conventional antidepressants for some patients. Moreover, analyzing the relationship between depression and inflammation suggests a future clinical application as it indicates the existence of an inflammatory subtype of depression. Within this subtype at least, a successful therapeutic intervention can be achieved. Moreover, it is possible that once the link between depression and inflammation is well established, it can serve as a new tool for detecting a biomarker and identifying a high-risk population. This will facilitate the management of stress and depression to prevent disease progression.

**Conflict of interest**: The authors declare no conflict of interest.

**Availability of data and materials:** All relevant data are within the manuscript and its Supporting Information files.

## Supporting information

S1 Table Primer sequences chosen for quantitative real-time polymerase chain reaction analysis. (PNG)

S2 Table Raw data for the effect of environmental paradigm in FST. (PNG)

S3 Table Raw data for the effect of pharmacological intervention in FST. (PNG)

S4 Table Raw data for RT-PCR experiments. (XLSX)
