# Supplementary figures and images for: Assessing the role of toll-like receptor in isolated, standard and enriched housing conditions
Source: PLoS One. 2019 Oct 24;14(10):e0222818. doi: 10.1371/journal.pone.0222818 (PMC6812767; doi:10.1371/journal.pone.0222818)

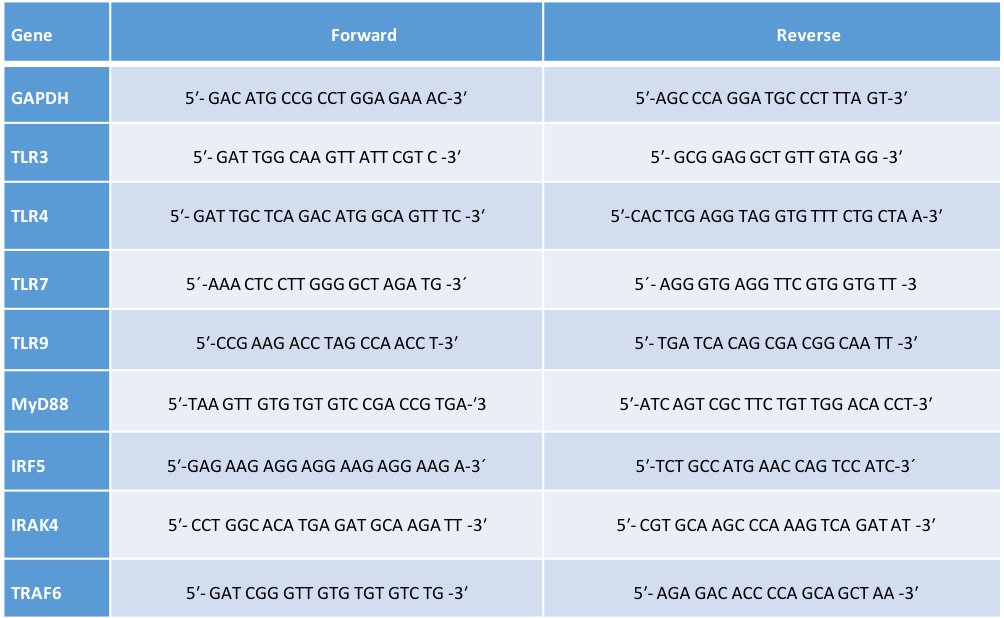

Supplement: S1 Table — (PNG) [file pone.0222818.s001.png]

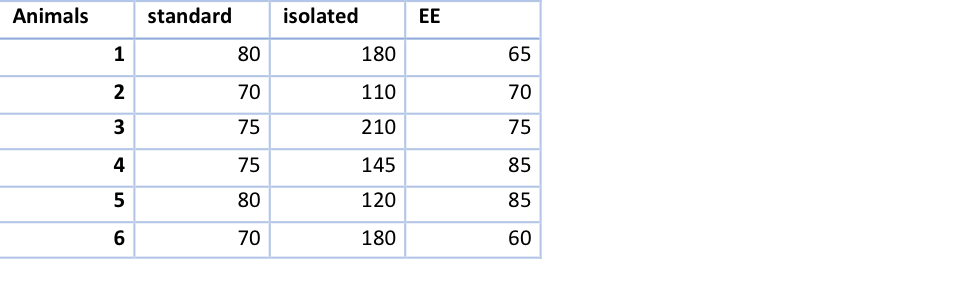

Supplement: S2 Table — (PNG) [file pone.0222818.s002.png]

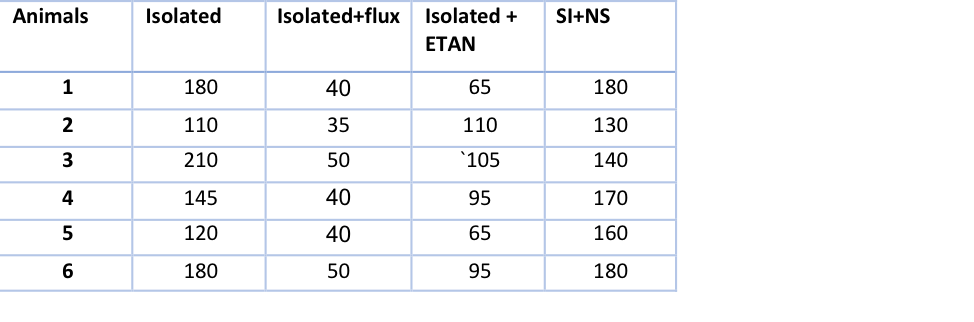

Supplement: S3 Table — (PNG) [file pone.0222818.s003.png]
